# E-book-based learning activity during COVID-19: engagement behaviors and perceptions of Japanese junior-high school students

**DOI:** 10.1186/s41039-022-00184-0

**Published:** 2022-03-21

**Authors:** Hiroyuki Kuromiya, Rwitajit Majumdar, Gou Miyabe, Hiroaki Ogata

**Affiliations:** 1 Graduate School of Social Informatics, Kyoto University, Kyoto, Japan; 2 Academic Center for Computing and Media Studies, Kyoto University, Kyoto, Japan; 3 Saikyo Junior High School Attached to Saikyo High School, Kyoto, Japan

**Keywords:** BookRoll, Learning and evidence analytics framework (LEAF), Technology-enhanced and evidence-based education and learning (TEEL) platform, Emergency remote teaching, K-12 education

## Abstract

Recent spread of the COVID-19 forces governments around the world to temporarily close educational institutions. In this paper, we evaluated learning engagement, level of satisfaction and anxiety of e-book based remote teaching strategy on an online learning platform. The research involves 358 students at an urban junior-high school in Japan. Learning logs were analyzed to measure student engagement, whereas survey responses indicated their perception regarding the remote learning experience. Log analysis revealed that the average completion rate over 267 learning materials was 67%. We also observed a significant decrease in engagement 3 weeks after remote learning and different subjects and grades. Survey analysis showed students felt both satisfaction and anxiety about remote learning. However, there were significant differences in the level of satisfaction between different grades. The results indicated that (1) maintaining students’ motivation is a challenge to remote learning in secondary schools, and (2) we need to relieve students’ anxiety about their own progress in the class and their classes after the break. This study is the first to report trends in actual teaching–learning engagement, which were recorded during sessions of emergency remote teaching in Japanese schools. The results can inform the future implementation of remote learning in junior-high schools.

## Background and motivation

Recent spread of the COVID-19 forces governments around the world to temporarily close educational institutions (UN, 2020). Faced with an inexperienced situation, teachers and schools were required to ensure that their students could continue learning at their home. It made teachers switch to emergency remote teaching (ERT) (Hodges et al., 2020). There were some review studies about ERT in higher and secondary education. Crawford et al. (2020) showed diverse responses by higher educational institutes over 20 countries: one group of universities did very little to respond and opted to meet the minimum standards, but one group rapidly closed their face-to-face classes and moved to remote teaching. ERT cases in secondary education were also reported by Bond (2020a, 2020b). The meta-review analysis suggested general challenges around the switch to ERT—teacher digital competencies, digital infrastructure, family access to technology, parent engagement in learning, and student health and well-being, etc. They also concluded that combining synchronous and asynchronous teaching is important for future remote teaching in K–12 education.

However, current studies pointed out there are two respects that are not sufficiently explored. One is quantitative analysis using students’ log data. According to Bond (2020a, 2020b), online surveys were the most used method for the analysis. Many studies lack the use of learning log data to validate remote teaching in schools. By combining log analysis and survey results, we can obtain a precise picture of the engagement behaviors of students in ERT. Another aspect is the case of a remote learning strategy based on e-books. Although e-book readers are common in remote learning, ERT studies using only e-books are scarce. In this paper, we analyzed 212 online teaching materials and 815,463 learning logs from 358 students during a period of conducting remote learning based on e-books while junior-high schools in Japan remained closed for face-to-face classes. We investigated the following two research questions:

1. What were the students’ engagement behaviors about e-book based learning activities during the school closure period?
2. What was the students’ perception of e-book based learning activity during the school closure period?

To answer these questions, we used both learning log data and survey responses of students.

Our paper is structured as follows. A review is presented on related remote teaching studies in higher and secondary education and the foundation of log analysis. Then, the context and learning tasks of ERT in target schools are described, including the collection of learning logs during the remote learning period. Next, we present the dataset and analysis procedure of survey data and students’ reading log data. The results are discussed in terms of their implication after a statistical analysis of the students’ engagement behavior and their perception. Finally, we explored the possibilities of our teaching strategies and the problems found by the analysis.

## Foundation of the study

### Emergency remote teaching strategies

According to Hodges et al. (2020), ERT is described as, “temporary shift of instructional delivery to an alternate delivery mode due to crisis.” Until now, many studies were published to handle this emergency situation in school and university. Nuere and de Miguel (2020) introduced two cases from different universities, one used to teach online and the other has no experience of it. They reported that while the university that was used to online teaching continued to use their own online learning platform, the university whose teaching is done in classrooms, they used their only ICT available tool, Moodle, and uploaded their materials online. However, they concluded that uploading materials online was not enough for online teaching because it lacks student-teachers interaction. Serhan (2020) reported the 31 cases where a web-based collaborative video conferencing tool, Zoom, was used in the class. The survey results showed that students’ had a negative attitude toward the use of Zoom and perceived it as having a negative effect on their learning motivation and experience. It indicates that the shift from face-to-face classes to remote learning was not easy neither for instructors nor for students who were not prepared. Lagi (2020) reflected the experience on the University of the South Pacific Tuvalu Campus. They reported that, even though Tuvalu is one of the COVID-19 free countries, the world pandemic situation affected their learning on campus by the decrease of the financial aid provided by 12 countries. These studies revealed a diverse reaction and teaching strategies in higher institutes to the COVID-19 pandemic.

Compared to ERT studies in higher education, studies in secondary education are not sufficiently investigated. Here, we will introduce several studies about ERT in schools. First, Bergdahl and Nouri (2021) explored the transition from traditional teaching into distance teaching in Swedish schools enforced by COVID-19. Survey to the teachers showed that the five most popular applications during COVID-19 crisis were Google Classroom, Google Meet, Youtube, Gleerups, and MS Teams. However, they also reported that the majority of the teachers had no strategy to implement distance education or less strategy to respond to a school closure. Some teachers reported that they had no support for using online teaching tools. Zhang (2020) reviews the educational system in China during the COVID-19 pandemic. They reported that remote teaching tools such as DingTalk (Alibaba), Tencent (WeChat application for assignment collection and grading or feedback) were common among teachers. However, they also reported that the teachers emphasized that online teaching–learning cannot replace face-to-face learning, and argued that remote teaching–learning only benefits those students with good self-discipline and high autonomy in learning. Niemi and Kousa (2020) described one local upper secondary school in Finland during the COVID-19 pandemic. In Finland, all teaching was changed to distance for 2 months. The study described that teachers mainly used MS Teams, but also other digital tools such as DVDs, Kahoot, Google Sheets, etc. After the perception survey to teachers and students, they concluded that distance teaching was implemented successfully, but students complained of heavy workloads and fatigue; some students lost motivation.

### Analyzing engagement from learning logs

To measure students’ engagement, we used e-book log data in the analysis. It can be seen as a “student-content interaction” in Moore’s interaction model (Moore, 1989). It is used to describe online learning during emergency remote periods (Abou-Khalil et al., 2021). Until now, several studies were published using students’ reading log data as a measure of the learning engagement. For example, Akçapınar et al. (2019) used the average completion rate of all books to predict students’ performance. Of course, we have several indicators to measure students’ engagement. For instance,  Kuromiya et al. (2020) used time spent on the materials as the learning engagement and compared the indicator to estimate the effectiveness of the intervention. However, we decided to use completion rate as students’ engagement because teachers made phone calls during the remote teaching period based on the students’ completion rate status. However, studies that use log data to measure students' engagement in the ERT period were less popular than normal periods. Majumdar,  Bakilapadavu, et al. (2021) describe a case on an e-book platform in Japan. They analyzed students’ reading behavior logs over 243 courses during the ERT period in a university and revealed that active learning actions such as use of annotations or use of memo was done more in humanities courses rather than engineering courses.

### Learning evidence analytics framework (LEAF)

**Fig. 1:**
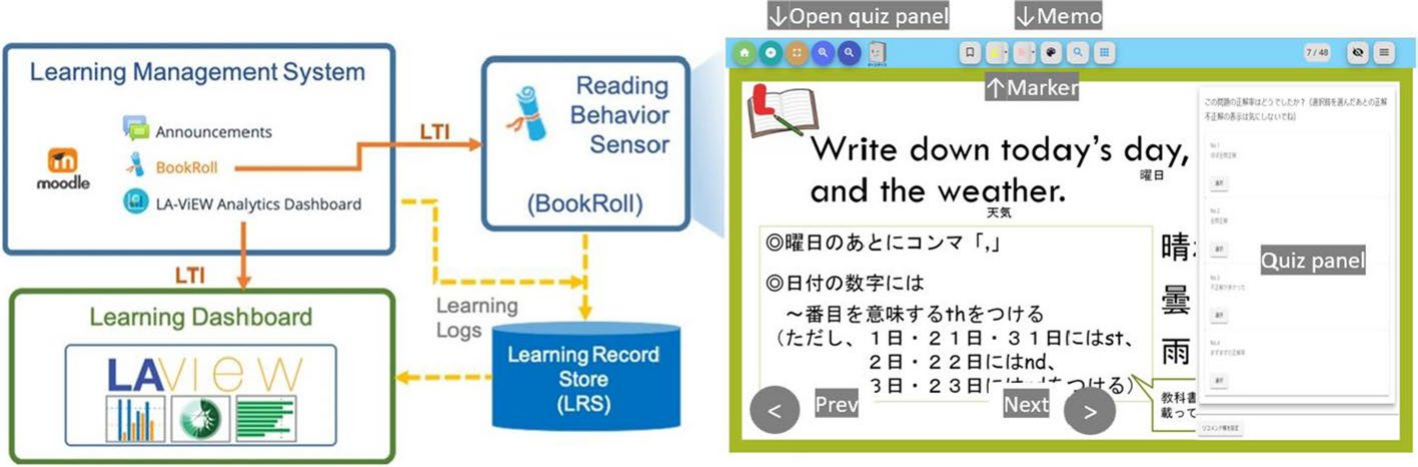
Architecture of LEAF platform

Ann integrated online learning platform was used in this study which instantiated the LEAF to a secondary school in Japan (see Fig. 1). LEAF is an overarching technology framework used to collect evidence of learning and teaching from logs generated within a technology-enhanced learning environment (Flanagan and Ogata, 2018). It consists of three different learning tools, Moodle, BookRoll, and LAViEW. The first component, Moodle, is a learning management system (LMS) used to host the courses, manage learning resources, and conduct assessments to evaluate students’ learning. The second component, BookRoll is an e-book reader, where teachers upload learning materials for students. BookRoll has many advantages compared to just uploading reading materials as a pdf format to Moodle. Teachers can create quizzes and recommendations on the material. Students can write handwritten memos, highlight text, and answer questions on any device which has a browser. Moreover, BookRoll can be considered a learning behavior sensor, because it can log students’ reading and annotation interactions in a Learning Record Store as statements of standard Experience API (xAPI). The third component, LAViEW, is the learning analytics dashboard that visualizes the learner interactions. Users can view the location of markers, memos, bookmarks, time spent on each page, and other reading behaviors as well.

## Study context and methods

### Target school and participants

The target school is an urban junior-high school in Japan. The study recruited 358 students with ages ranging from 12 to 15 years; males were 142 (39.7%) and females were 216 (60.3%).

In Japan, the first peak of COVID-19 cases came in April 2020.^1^ In the target school, requested from the city government, the school principal decided to temporarily close the school from 10 April to 12 June, 2020. For the first month, teachers distributed a health check survey and small assignments every day on Moodle, but after 1 month later, they started a specific remote teaching activity conducted on an e-book platform. The last 2 weeks were the “warming-up” period for the school, half of the students going to the school on 1 day and the other half did self-study using the shared materials on BookRoll. They switched to coming to school every alternate day. The target period for the analysis of learning logs was set from May 11 to June 12, 2020. In total, there were 5 weeks during the target period. The subsequent section describes remote teaching in the target school in detail.

### E-book based remote teaching strategy

**Fig. 2:**
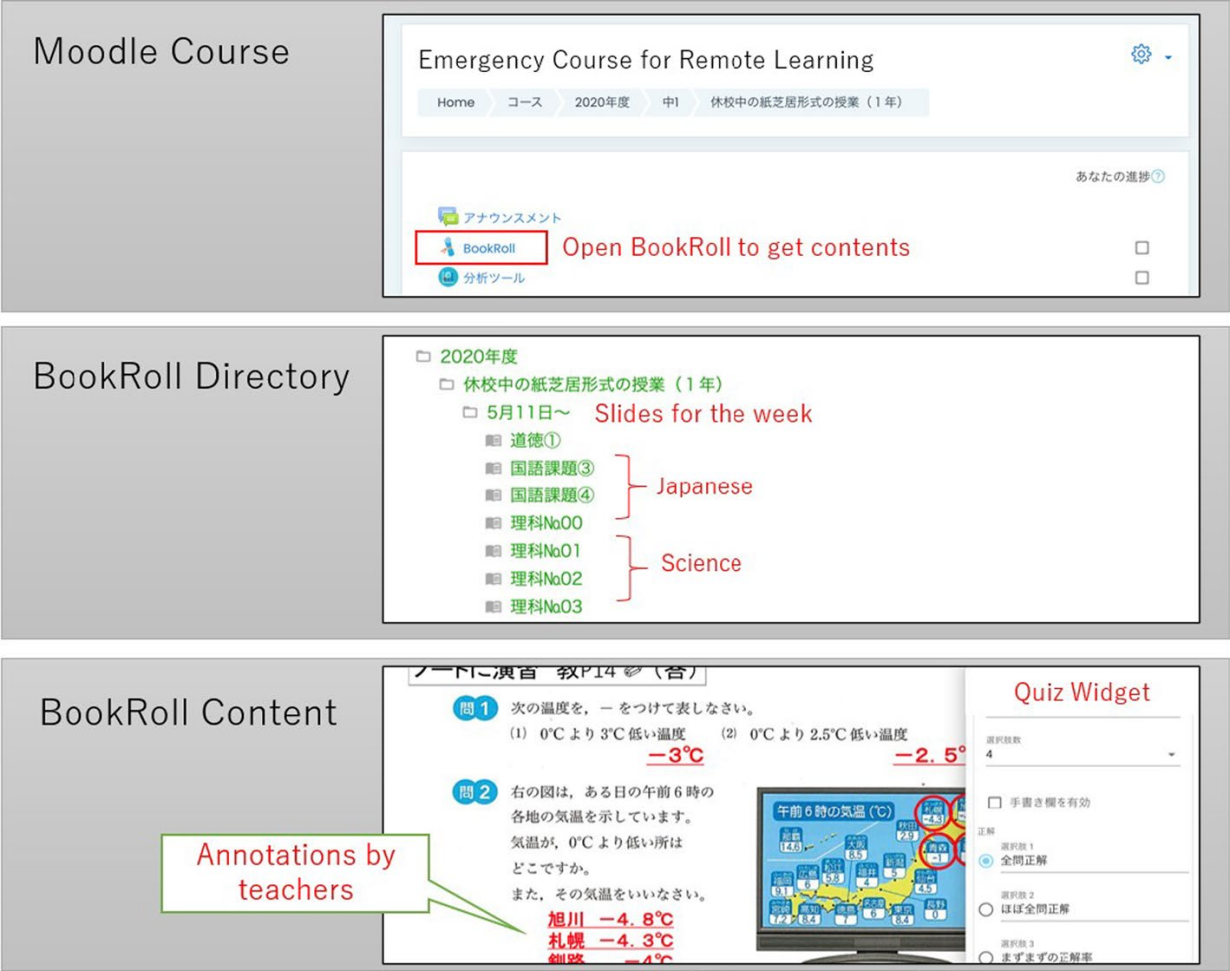
Screenshots of the e-book based remote learning strategy on the LEAF platform

Here we introduce the slide-based instruction on an e-book learning platform and its workflow that was adopted during ERT at the target school. Remote learning activities were conducted on the LEAF platform (see Fig. 2). The media used to disseminate assignments were the Moodle assignment/questionnaire module, BookRoll quiz and memo, or submission of papers. Teachers prepared one course for the school closure period by each grade and uploaded all the materials in one folder in BookRoll. It made students find the contents easy. The learning content includes the essence of usual face-to-face classes and the important sections of a textbook. It also instructs students to take note and solve the problems in the textbook, which should be instructed in face-to-face classrooms.

The following list presents the workflow of the e-book based remote learning from the perspective of the students.

1. Received learning materials for 1 week (20 h) every Monday morning via the LEAF platform.
2. Created a self-study plan for 4 h per day. Students are given the freedom to decide which subject to do on which day of the week; and
3. Followed the instructions and assignments included in the materials.

The students were expected to self-study for 20 h per week. Although school time can reach 32 h per week in Japan, the teachers’ consensus indicated 20 h given the time capacity of junior-high school students for self-management. Prior to implementing e-book based remote teaching, we conducted two sessions of teacher training. The first explained the outline of the implementation during the closure period, such that the policy requirements of the education board regarding the number of learning hours at home is fulfilled. The second was held 1 week prior to ERT to confirm the plan regarding the number of sheets, contents, and delivery method of ERT through the LEAF platform.

## Data collection and analysis

In the e-book based remote learning at the target school, 267 learning contents were distributed on BookRoll during the school close period. However, we excluded 12 materials because they were just notifications to students instead of learning contents. Also, we filtered the materials based on their subject. This study only targeted five main subjects—Japanese, English, Math, Science, and Social Studies; thus, 43 materials unrelated to these subjects were excluded. Finally, we got 212 materials as target contents. Average pages in those contents were 19.3 pages (SD: 12.6) in the obtained dataset. For the analysis of learning behaviors, we collected the learning logs of students related to these contents.

### Learning logs

**Fig. 3:**
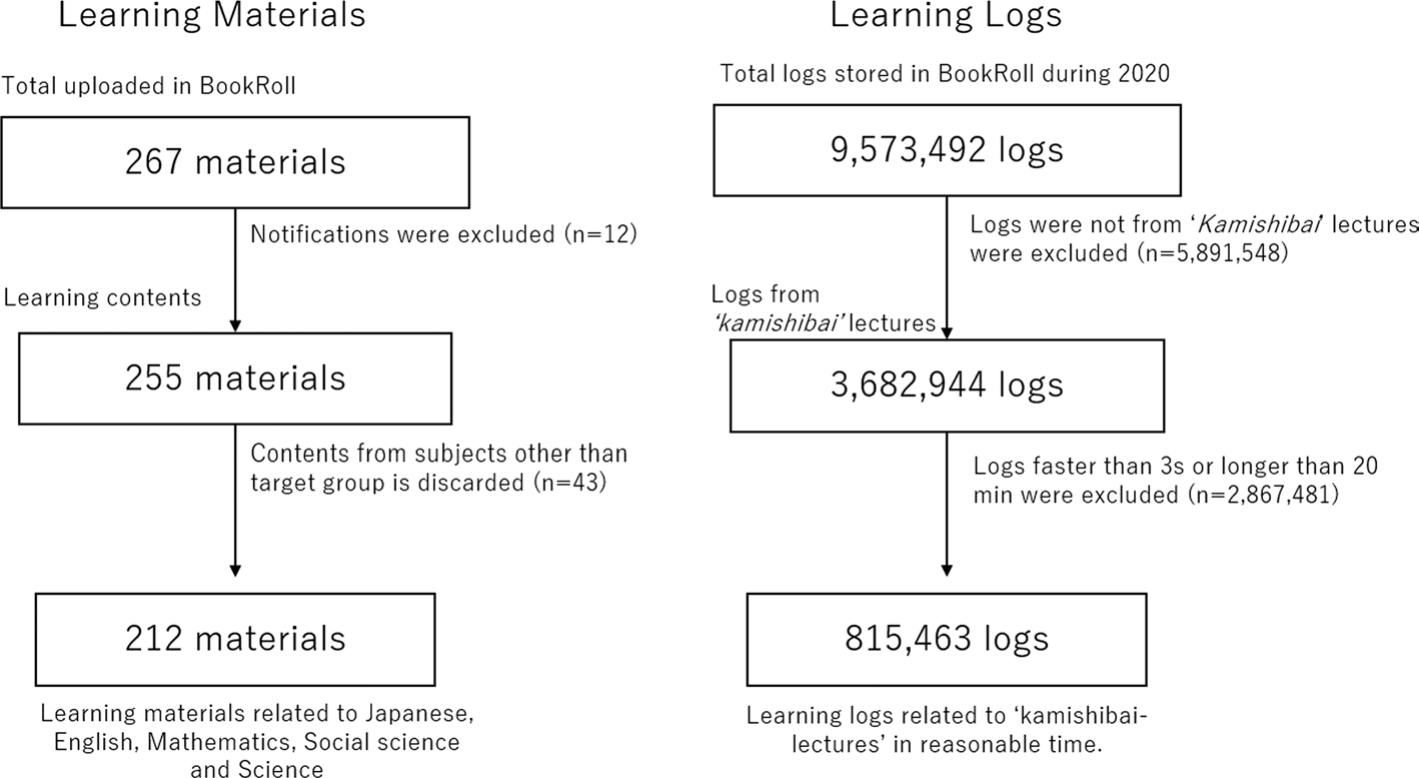
Dataset filtering criteria

To measure students’ engagement on that material, we analyzed students’ reading log data on BookRoll. In the last year, we had 9,573,492 logs in total. In that, 3,682,944 logs were of the target contents, but we excluded 2,867,481 logs because students’ operation was too quick (less than 3 s) or too long (longer than 20 min), which implies students’ off-task behavior on the material. The thresholds were determined by the previous studies on e-book based learning interactions (Majumdar, Bakilapadavu, et al., 2021a, Majumdar, Flanagan, et al., 2021b; Yin et al., 2019). Finally, we included 815,463 logs for our analysis (Fig. 3). Based on the logs, we calculated students’ average completion rate as the indicator of learning engagement.

For analysis of the learning logs, an overview of students’ engagement by school year, subjects, and learning weeks were visualized. There were three different school years (Junior-High (JH) 1, 2, 3), five subjects (Japanese, English, Math, Science, and Social Studies), and five learning weeks (week 1–5). An analysis of variance (ANOVA) was conducted to check if there were any significant effects on reading completion by weeks, grades, and subjects. JASP statistical analysis software (Love et al., 2019), version 0.14.1, was used for the purpose.

### Perception survey

Aside from the learning logs, we collected survey responses to evaluate the students’ perception of e-book based remote teaching during the school closure period. The survey was created based on a discussion with the teachers during the training sessions. Questions No. 1–4 were evaluated on a point scale (strongly agree: 6, moderately agree: 5, slightly agree: 4, slightly disagree: 3, moderately disagree; 2, strongly disagree: 1). Questions No. 5–7 were yes/no questions, and Question No. 8 and 9 were multiple-choice questions, which contains 6 sub-items for each. All question items were listed on “Appendix”. The survey was collected via Moodle feedback module. These responses were mandatory for students and had to be filled in by 15 June.

To compute the average satisfaction and anxiety level of students, we first analyzed questions No. 1–4 because they were the only Likert scale questions in the survey. One-sample one-sided T-Test was conducted for each item. Test value was 3.5 because it was the midpoint of the scale. After that, question No. 9 and its subitems were analyzed for revealing students’ concrete anxiety content. For the statistical testing, JASP (Love et al., 2019) version 0.14.1 was used in the survey analysis, too. Descriptive statistics for other questions are shown in “Appendix”.

## Results and interpretation

### Insights from log data analysis

**Fig. 4:**
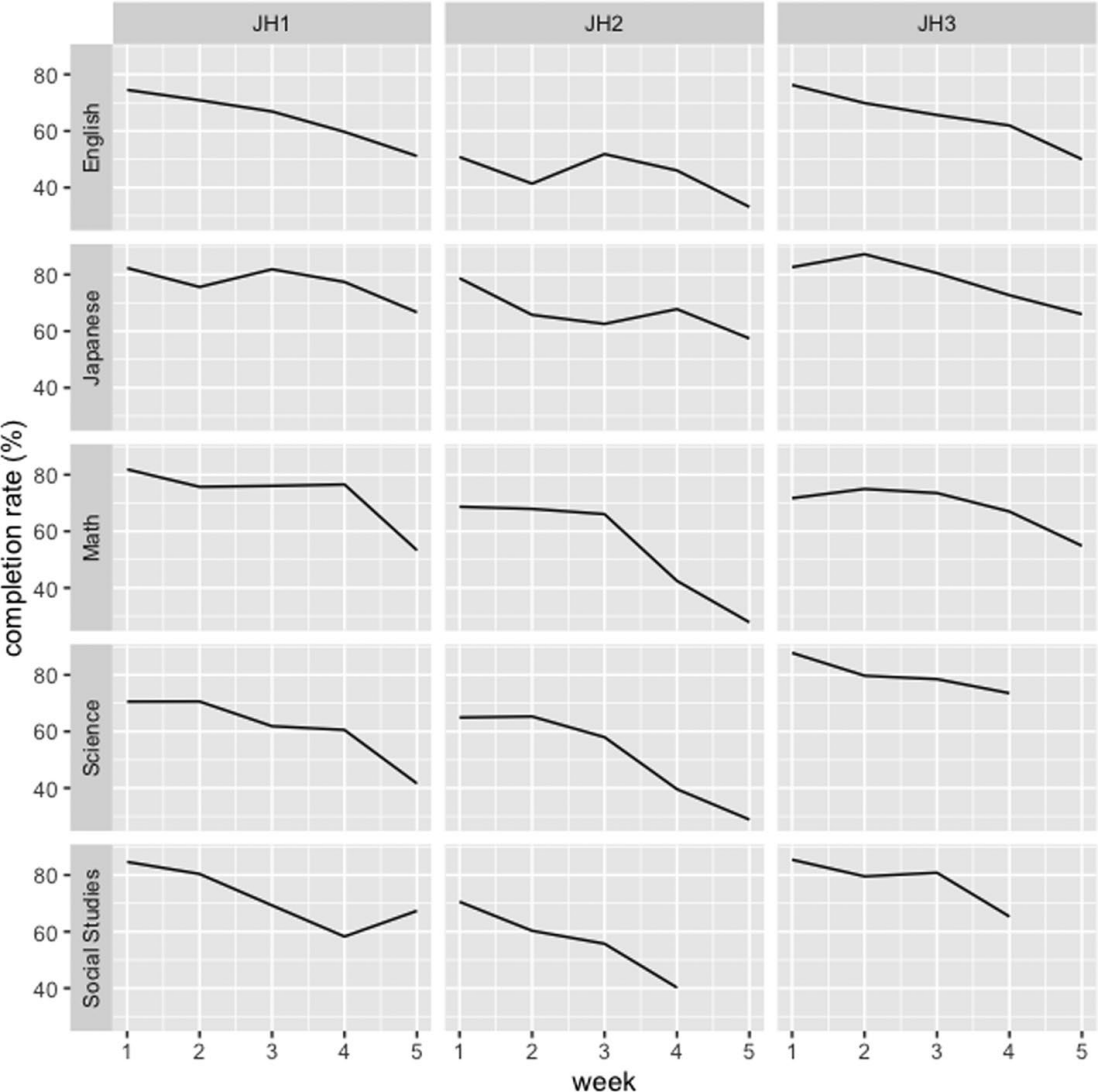
Average completion rate by school year, subjects, and week

The overall average completion rate during the remote learning period was 67.0% (SD: 13.97%). To take a closer look, we plotted the dynamics of average completion rate by school year and subjects in Fig. 4. In the figure, we can see that (1) all of the lines have decreasing trend, (2) overall, the values in JH2 were lower than other grades, and (3) there were some gaps among subjects. To check these three trends, we conducted ANOVA to test significance.

First, we tested if there is a significant effect by weeks on completion rates. As we could not confirm the equality of variance (*p *= .03), we conducted ANOVA test with homogeneity corrections (Brown-Forsythe). As a result, we observed a significant effect by weeks (*p *< .001, *df *=4 ). Therefore, we went through the post hoc comparisons by each week. Table 1 shows the difference of average completion rate from the first week of the vacation. A post-hoc Tukey test showed that there were significant differences between students’ engagement on week 1 and week 3–5. The results indicates that student engagement has declined significantly after week 3.

**Table 1:** Average completion rate by week

	Average completion rate (%)	Difference from week 1 (%)	*p*
Week 1	74.8	–	–
Week 2	70.7	− 4.06	.42
Week 3	67.7	− 7.05	.02
Week 4	60.4	− 14.3	< .001
Week 5	49.0	− 25.7	< .001

Also, we tested if we observed any significant effects on completion rates by grades and subjects (see Table 2 for descriptive statistics). We conducted two-way ANOVA and found significant relationships from grades (*p *< .001, *df *= 2) and subjects (*p *< .001, *df *= 4). Post hoc Tukey comparison by grades showed a significant drop in engagement in the second grade. The difference between JH1 and JH2 was 13.9 % (*p *< .001) and JH2 and JH3 was 16.4 % (*p *< .001). On the other hand, the difference between the first grade and the third grade was not significant (*p *= .43). Moreover, we tested if there were any significant differences between subjects. Japanese was the highest engagement subject and English was the lowest. The post hoc Tukey comparison showed that English was significantly lower than Japanese (*p *< .001), Math (*p *= .002) and Social studies (*p *< .001) and Japanese was significantly higher than Science (*p *= .002).

**Table 2:** Average completion rate by grade and subject

	English (%)	Japanese (%)	Math (%)	Science (%)	Social studies (%)	Mean (%)
JH1	67.5	77.9	76.4	62.6	72.3	70.5
JH2	45.9	68.6	57.3	53.9	58.2	56.7
JH3	66.4	79.0	70.2	78.6	78.7	73.0
Mean	60.5	75.0	68.6	62.0	69.7	67.0

Therefore, to answer the research question 1, we could say these three findings regarding student engagement behaviors: (1) the overall average completion rate was 67 %, (2) The student engagement as interpreted by the learning logs had a downward trend 3 weeks after, and (3) students in the second grade were less engaged than other grades.

### Insights from survey analysis

**Fig. 5:**
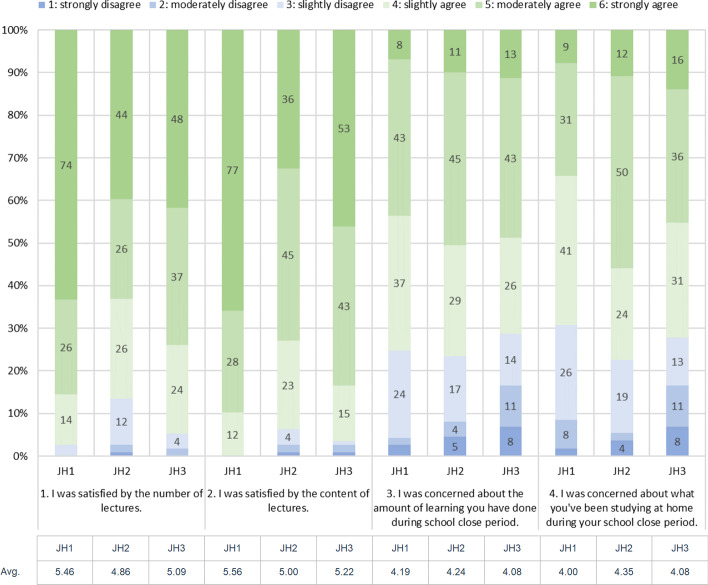
Survey responses by grades

The response rates for the survey were 97.5% for JH1, 94.1% for JH2, 95.0% for JH3. Cronbach’s α over the all four items was 0.66. Figure 5 shows the distribution of the responses over the four items. Responses in favor are shown in green, while those against are shown in blue. Chi-square tests were significant in Q1 (*p* < 0.001), Q2 (*p* < 0.001), Q4 (*p* = 0.004), and not significant in Q3 (*p* = 0.10). The average point values were M: 5.14 (SD: 1.01), M: 5.25 (SD: 0.91). M: 4.17 (SD: 1.23), and M: 4.14 (SD: 1.24) for each question (see “Appendix” for details). These results suggest that students predominantly felt both satisfaction and anxiety about the distance learning during the school holidays. However, when the satisfaction levels were compared between the grades, there was a significant difference between the grades. Post hoc Tukey test showed that there were significant differences between JH1 and JH2 (*p* < 0.001) and JH1 and JH3 (*p* = 0.01) in Q1, and between JH1 and JH2 (*p* < 0.001) and JH1 and JH3 (*p* = 0.01) in Q2. There was no significant difference in the level of dissatisfaction between the grades for either Q3 or Q4.

**Fig. 6:**
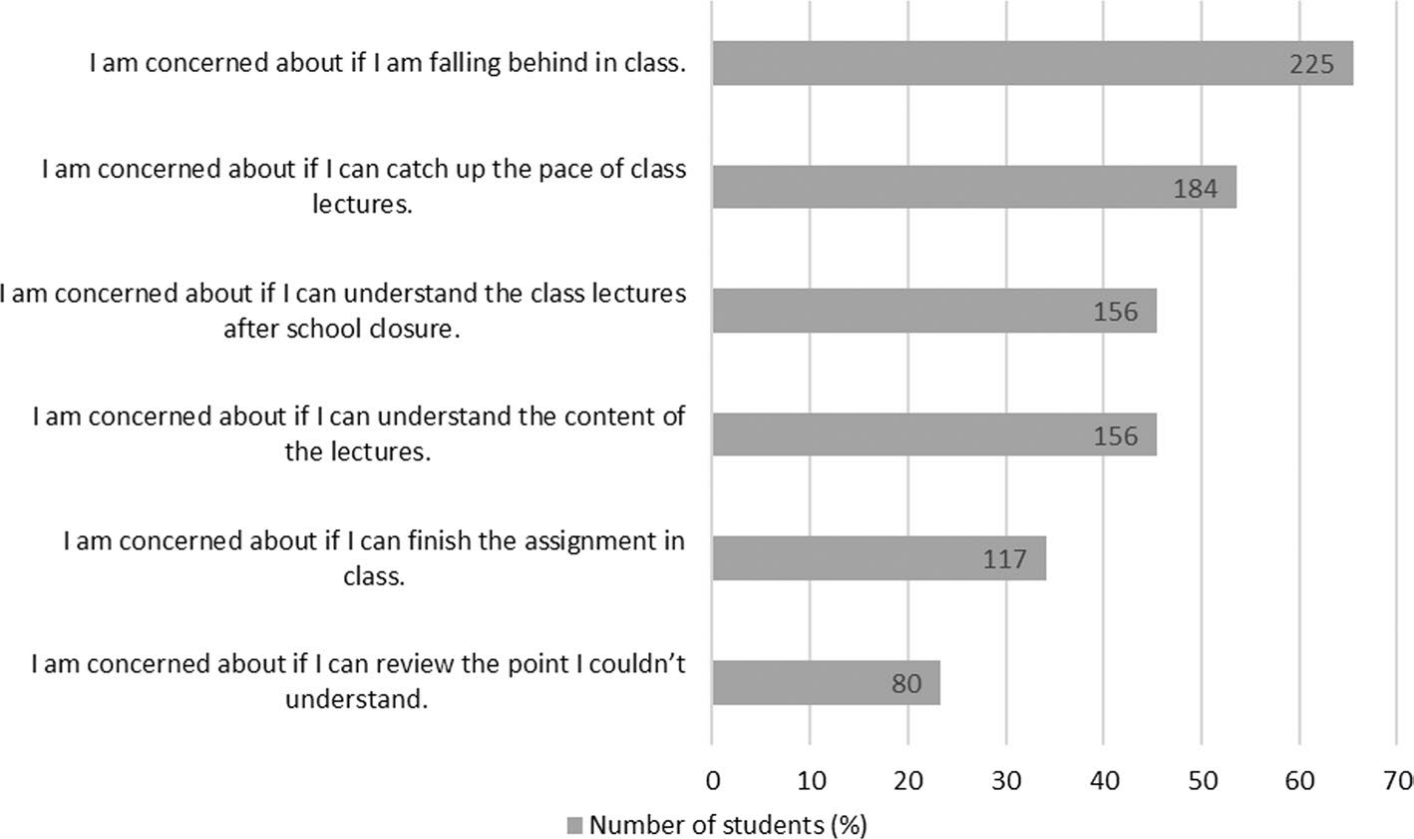
Students’ concerns about remote learning

In summary, to answer the research question 2, first-year students were highly satisfied with and confident about remote learning during COVID-19 compared with the other grades. In addition, the first-year students felt that they possessed good self-direction skills in terms of learning at home. Among the three grades, the second-grade students were the least satisfied with remote learning, which is consistent with the result of learning engagement analysis based on learning logs. According to Fig. 6, we can say that the main content of students’ anxiety was about the pace of learning. Over the half of the students felt like “I am falling behind in class” or “I couldn’t catch up the pace of the class.” Unlike face-to-face classes, it is difficult to see the progress of others in remote classes, which may have led to this result.

## Discussion

### Analyzing learning log data for capturing emergency teaching period

Our study revealed the importance of analyzing actual log data to understand the real situation in the ERT period. As Bond (2020a, 2020b) mentioned, currently, the most popular way to investigate students learning in ERT is online surveys. However, it does not always reflect the actual status of students. In our situation, although survey results supported students’ satisfaction toward remote learning, we observed significant engagement decrease from log data. Therefore, we encourage researchers to investigate learning logs as well as the survey in order to make the situation clear during the ERT period. Our study confirms the applicability of e-books in e-learning practice.

This study provides analysis from actual data collected from a public school in Japan. It is useful as a baseline for interested stakeholders to refer both domestically in Japan and also in a global context for compulsory public education (junior-high school level in Japan). As described in Fig. 3, other schools can adopt the current method for ERT if necessary, in these uncertain times. In order to improve the emergency remote teaching in our e-book platform, we will discuss the following two points based on the results of this analysis.

### Factors that affected the difference in engagement of emergency learning

To substantiate our findings from log analysis, we investigated previous research evidences to explain the results. First, decreasing students’ motivation is a well-observed phenomena in online learning (Heyman, 2010). Therefore, we expect that similar approaches can be taken for dealing with this issue by referring to previous studies. For example, Bawa (2016) suggested four possible solutions to retain students’ motivation in online class—1. making orientation programs mandatory, 2. using “live” interaction and transparency in computer mediated communication (CMC), 3. creating classes structured for collaborative learning, and 4. enhancing faculty training support.

In our context, the e-book based remote teaching did not involve interactive teaching sessions nor synchronous collaborative learning as mentioned in the listed item 2 and 3. Therefore, it is highly likely that the points listed in 1 or 4 above may also have affected the differences in engagement between grades and subjects. Regarding the point 1, the school had a basic orientation program to all the students before closing the school. It has been pointed out that students tend to be resistant to this kind of training as unnecessary (Bozarth et al., 2004), but younger students may have been less resistant to it. Regarding the point 4, we did not organize a training session on remote teaching methods before this emergency period. However, it is highly likely that the presence of a leader in the use of the e-book platform in the first year of junior high school would have enhanced the ICT skills of the teachers in the same grade. The other grades did not have such a leader teacher.

### Factors that affected the students’ perception emergency learning

For the interpretation of the results of the survey, we introduce some studies that have asked learners about their satisfaction with emergency remote learning. For example, a study conducted in Malaysia using a similar questionnaire also reported that students showed positive feelings towards distance education during COVID-19 (Yuan, 2021). Here, the authors asked students' satisfaction in the point of teaching materials, assessment, communication, and technical support, all of which were highly satisfactory. The fact that similar positive results were obtained in studies with completely different contexts may be due to acquiescence bias, which makes respondents more likely to answer “yes” to a question (Baron-Epel et al., 2010). In our study, the questionnaire was developed along with consensus of teachers and kept easy to comprehend for the target population. However introducing inverse items would have enabled us to adjust acquiescence effect though increasing the number of items in the questionnaire.

On the other hand, there is a study that focused on students' “dissatisfaction” in distance learning by Tulaskar and Turunen (2021). They conducted a questionnaire survey and interview with students in Finland and India, and found that scheduling, distractions, pessimistic emotions, longer durations, and maintaining concentration were highest challenges faced by the students during the emergency learning period. In our context, students were dissatisfied not with the content or quantity of the assignments, but with the fact that they don’t know how to pace themselves and that they may not be able to keep up with the classes after the break. Therefore, in order to alleviate students' anxiety, the Learning Analytics dashboard could be used to show them what other students in the same class are working on, or to show them a concrete plan for classes after the break.

## Limitations and future work

Thus far, we have introduced our overview of ERT in a Japanese junior-high school from the perspectives of behavior and survey analyses. Through the analysis, we could understand what kind of teaching was done in the target school and how students perceived it in terms of learning engagement and concerns toward remote learning. However, the study has its limitations. In behavior analysis, we only targeted the completion rate of students in terms of contents. However, several other metrics can be used to gauge the learning engagement of e-book readers in general (Akçapınar et al., 2019) and specifically during ERT period (Majumdar, Bakilapadavu, et al., 2021a; Majumdar, Flanagan, et al., 2021b). In particular, the school teacher pointed out the impact of differences in the density of teaching materials in different subjects. For example, the content of each page is rich in Japanese, students may have been staying for a long time. On the other hand, English is made with the feeling that the content of each page is not so much and the slides proceed at a good tempo. The completion rate is not considered unless the user stays less than 3 s, so materials that make the user turn the page at a fast pace may have a lower view completion rate than actual.

The next limitation is the reliability of the survey, which was formulated by the teachers based on their interest. In other words, the survey lacks theoretical background in its items. Thus, the need emerges to create a survey on students based on previous surveys that used various templates about ERT in school. Moreover, there are some important questions that we could not answer in this paper (e.g. what kind of content design was effective? or what caused the difference among grades). We will continue to analyze the dataset and answer the questions above as the next study.

## Conclusion

In this paper, we verified learning behaviors and perceptions about an e-book based remote teaching strategy on an online learning platform at a junior-high school in Japan. Analysis suggested significant differences in completion rates and perception across the grades. Moreover, the results indicated that (1) maintaining student motivation is a challenge to remote learning in secondary schools and (2) we need to relieve students' anxiety about their own progress in the class and their classes after the break. This is the first case where ERT in Japanese secondary schools was described in academic papers. We hope that the results will contribute to and inform the implementation of future online learning in junior-high schools.

## Data Availability

Not applicable.

## Appendix: Survey items and responses summary

**Table:**

	Item type	Mean (SD)	Selected students (%)
1. I was satisfied by the number of lectures	6-point scale	5.14 (1.01)	–
2. I was satisfied by the content of lectures	6-point scale	5.25 (0.91)	–
3. I was concerned about the amount of learning you have done during school close period	6-point scale	4.17 (1.23)	–
4. I was concerned about what you've been studying at home during your school close period	6-point scale	4.14 (1.24)	–
5. I was able to plan study schedule every week	Binary (yes/no)	–	232 (67.6%)
6. I was able to open lecture materials every normal day	Binary (yes/no)	–	282 (82.2%)
7. I was able to study at home for 2-h every day	Binary (yes/no)	–	262 (76.4%)
8.1. I could understand the content of the lectures	Multiple-choice item	–	256 (74.6%)
8.2. I could catch up the pace of the lectures	Multiple-choice item	–	204 (59.5%)
8.3. I could do the assignment in the lectures	Multiple-choice item	–	210 (61.2%)
8.4. I could continue studying as planned	Multiple-choice item	–	160 (46.6%)
8.5. I could understand what I should study after finishing the 20-h content of the week	Multiple-choice item	–	82 (23.9%)
8.6. I could understand what I should study in self study	Multiple-choice item	–	117 (34.1%)
9.1. I was concerned about if I can understand the class lectures	Multiple-choice item	–	156 (45.5%)
9.2. I was concerned about if I can catch up the pace of class lectures	Multiple-choice item	–	184 (53.6%)
9.3. I was concerned about if I can finish the assignment in class	Multiple-choice item	–	117 (34.1%)
9.4. I was concerned about if I can review the point I couldn’t understand	Multiple-choice item	–	80 (23.3%)
9.5. I was concerned about if I am falling behind in class	Multiple-choice item	–	225 (65.6%)
9.6. I was concerned about if I can understand the content of the lectures	Multiple-choice item	–	22 (24.5%)

## Abbreviations

COVID-19: Coronavirus disease 2019
ERT: Emergency remote teaching
LEAF: Learning and evidence analytics framework
xAPI: Experience API
JH1: Junior-High School Grade 1 Students
JH2: Junior-High School Grade 2 Students
JH3: Junior-High School Grade 3 Students
ANOVA: An analysis of variance

## Footnotes

1 http://japan.kantei.go.jp/ongoingtopics/_00013.html
